# A Super Memory Processing Unit Based on 3D Stacking and Hybrid Bonding for High-Efficiency AI Computing

**DOI:** 10.3390/mi17070802

**Published:** 2026-06-30

**Authors:** Ruiyong Zhao, Yibo Hu, Jing Chen

**Affiliations:** Shanghai Institute of Microsystem and Information Technology, Chinese Academy of Sciences, Shanghai 200031, China; zry@mail.sim.ac.cn (R.Z.); ybhu@mail.sim.ac.cn (Y.H.)

**Keywords:** processing in memory (PIM), DRAM, memory bandwidth, multi-modal large language model

## Abstract

DRAM-based in-memory computing integrates computational regions into the main memory, enabling local data processing within the memory, thereby achieving faster and more efficient data computation. However, enhancing system performance requires addressing a critical challenge: achieving more general and sufficiently powerful data processing capabilities within DRAM-PIM. Existing DRAM-PIM implementations often suffer from limited computational capabilities due to the shared standard DRAM package area between memory cells and computational circuits or because the operator circuits are overly customized, which limits their ability to meet required data processing demands. To address this issue, in this paper, we propose a Super Memory Processing Unit (SMPU). The SMPU uses Hybrid Bonding technology to 3D-stack DRAM and many-core computational clusters, enabling large-bandwidth (0.25 TB/s per-bank, 2 TB/s for 8-bank system bandwidth) on-chip data transmission between DRAM and the computational cluster via copper interconnects, effectively breaking the memory wall bottleneck of existing computing architectures. The SMPU constructs a dual-channel fine-grained computational cluster at the logical computing layer, providing flexible and ample computility for various AI models, such as ResNet50 and Llama2. The SMPU uses standard DDR protocols and integrates a new memory space allocation and parsing controller to ensure system compatibility without modifying the host-end hardware, facilitating the integration and invocation of computility in memory particles. Additionally, the SMPU features an independent dual-channel memory-management mechanism within the memory particles, enabling simultaneous multi-channel, multi-modal AI model inference. We compared a CPU system equipped with an SMPU to current computing systems using FPGA simulations. The FPGA simulation results show that, under the same computational configuration, the system with the SMPU improves the performance of ResNet50-v1.5 by up to 5.1× and Llama by up to 27.43× compared to the base system, while reducing system power consumption by 71.6% (ResNet50-v1.5) to 77.8% (Llama 7B).

## 1. Introduction

AI models have become a research hotspot in the field of artificial intelligence, particularly in applications such as natural language understanding and generation, machine translation, dialog systems, and text summarization [1,2,3,4]. High-performance computing chips face three major challenges when handling AI tasks: high demand for computational resources, energy consumption and efficiency issues, and memory access bottlenecks. AI tasks often require the simultaneous processing of multiple data types, including visual, auditory, and textual data, necessitating strong parallel computing capabilities and high memory bandwidth to support the concurrent processing of multi-modal data. This ensures that models can operate efficiently in complex scenarios, providing accurate and rapid responses [1].

Despite the significant increase in computational resource requirements, traditional GPUs and other accelerators may struggle to meet these needs, especially in real-time processing scenarios. Additionally, the processing of complex AI tasks leads to increased energy consumption, affecting the energy efficiency and thermal management of computing chips. This is particularly critical in mobile devices and edge computing environments, where energy efficiency is a key consideration [2]. Furthermore, AI tasks involve extensive intermediate feature storage and exchange, placing higher demands on memory access patterns. Traditional memory architectures may not efficiently support the fast read/write operations required to handle large numbers of features, leading to performance bottlenecks. These challenges collectively limit the effectiveness and applicability of current computing chips in AI task processing.

**Figure 1:**
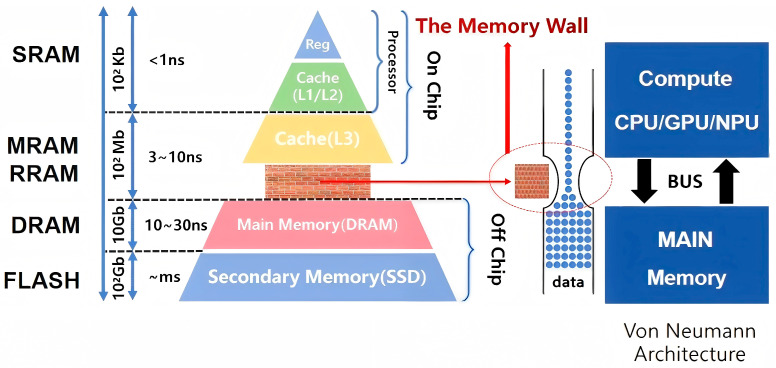
The memory wall in Von Neumann architecture.

Currently, AI-specific processing chips are almost universally constrained by memory bandwidth bottlenecks, as shown in Figure 1, which severely limit the efficiency of model training and inference. AI tasks require the processing of large amounts of parameters and data, demanding extremely high memory bandwidth, which existing computing architectures often fail to provide [2]. Moreover, memory access latency is a significant issue, as it increases waiting times during computation, thereby affecting overall processing speed. To mitigate this bottleneck, researchers are exploring various solutions, including improving memory subsystem architecture design (such as SRAM-based in-memory computing and HBM-PIM technologies), adopting higher-bandwidth memory technologies (such as HBM in 2.5D packaged SoCs), optimizing data access patterns, and developing hardware accelerators specifically for AI models. However, these methods still face numerous challenges in practical applications, such as cost, power consumption, and compatibility. Therefore, finding a solution that can effectively enhance memory bandwidth while maintaining a good cost–performance ratio has become a key focus of current research.

With the rapid iteration and development of AI models, the parameter scale has reached hundreds of gigabytes, making DRAM the most suitable storage medium among currently mature industrial storage materials to accommodate such large-scale model parameters [5,6,7,8,9,10,11,12,13]. Therefore, DRAM-PIM is considered the most advantageous PIM architecture for overcoming memory wall bottlenecks, offering better computational performance compared to SRAM and other storage media-based PIM. However, previous DRAM-PIM implementations have been limited by the shared standard DRAM packaging area between memory cells and computational circuits or overly customized operator circuits, which restrict their ability to meet data processing demands. To address these issues, we propose a Super Memory Processing Unit (SMPU).

The SMPU aims to overcome the memory wall and power wall bottlenecks faced by AI processing chips while ensuring the versatility of deployed models. It employs 3D-stacking technology to bond DRAM wafers with logic computing wafers face-to-face, using Hybrid Bonding copper interconnects to directly connect the DRAM storage layer wafer with the many-core compute cluster layer wafer, creating a high-speed, wide data bus for intra-chip data transmission between storage and compute clusters, achieving ultra-high-bandwidth (2 TB/s) data transfer. This design effectively breaks the memory wall bottleneck in existing computing architectures. Additionally, SMPU particles are compatible with the standard DDR protocol, ensuring system compatibility without modifications to the host interface, thus seamlessly integrating with existing intelligent computing ecosystems. The in-memory computing chip within SMPU introduces a new memory controller that enables synchronous data transfer (both intra-chip and extra-chip) and in-memory computation, achieving extremely high data processing capabilities within the DRAM.

For model deployment on SMPU, we adopt global weight stasis and algorithmic hybrid parallel techniques. Weight data are stored in a dedicated memory address space within the SMPU’s PIM, remaining static during inference or training, with only values updated, minimizing data movement within the memory particle. Different deployment strategies are used for different model structures. For CNN models with high computational demand and low memory bandwidth requirement, both local and global data parallelism are employed. For Transformer models with lower computational demand and higher memory bandwidth requirement, a hybrid parallel deployment approach is used, combining local data parallelism with global operator parallelism. To validate the correctness of the SMPU architecture before fabrication, we conducted FPGA simulations. In our evaluation, the host processing platform (XPU) comprises a general-purpose CPU and a dedicated Neural Processing Unit (NPU). We compare a traditional architecture (XPU + 2 × DRAM) and an SMPU architecture (XPU + DRAM + SMPU). The simulations aimed to evaluate AI task performance in the following scenarios:

- In the traditional architecture, data is continuously transferred from DRAM to XPU via the LPDDR4 interface for computation and result return.
- In the SMPU architecture, the majority (50% to 70%) of linear computation tasks are directly completed within the SMPU chip.

The performance of these two architectures was subsequently compared to assess the effectiveness of the SMPU in improving AI task processing efficiency. FPGA demo results showed that under the same computational configuration, the system with the SMPU improved the performance of ResNet50-v1.5 by up to 5.1× and Llama by up to 27.43× compared to the base system, while reducing system power consumption by 71.6% (ResNet50-v1.5) to 77.8% (Llama 7B). This study supports a memory computing chip with a standard DDR protocol interface. The contributions of the proposed architecture include the following:

- Using Hybrid Bonding 3D-stacking of DRAM and a many-core computing architecture to truly break the memory wall, significantly enhancing the performance of computing systems in AI task processing.
- Employing a PIM architecture to fully perform multi-channel, multi-modal, and high-efficiency AI model inference within the memory particle.
- Adopting a standard DDR protocol interface to ensure seamless compatibility with existing computing ecosystems, requiring no major modifications to the motherboard or processor, ensuring low cost and low power consumption.

## 2. Background

### 2.1. AI Models and the Memory Wall Bottleneck

In recent years, the rapid advancement of deep learning has driven the widespread application of artificial intelligence (AI) across various domains, including computer vision, natural language processing (NLP), speech recognition, and autonomous driving. Particularly, the rise of deep neural networks (DNNs) has led to significant breakthroughs in tasks such as image classification, object detection, machine translation, and dialog systems. However, as AI models continue to deepen and expand, their increasing demands for computational resources and memory bandwidth have made the memory wall a critical bottleneck in performance enhancement.

AI models, especially DNNs, have become the core of modern AI technology. These models simulate the structure of human neurons through multiple layers of nodes and connections, with each node storing a value and undergoing forward and backward propagation for computation and optimization. In recent years, the scale of AI models has grown rapidly; for example, NVIDIA’s Megatron model contains 8.5 billion parameters, Microsoft’s Turing-NLG model has 17 billion parameters, and Open-AI’s GPT-3 model boasts 175 billion parameters. These large-scale models not only require more computational resources but also demand greater memory capacity and higher memory bandwidth to support their training and inference processes. Consequently, the memory wall is a fundamental limitation in modern computing architectures for handling AI models. The primary aspects of the memory wall problem in the context of AI models include the following:

- Bandwidth Limitation: Traditional memory systems, such as DRAM, have limited bandwidth, which can become a bottleneck when transferring large amounts of data between memory and computational units. For large-scale AI models, high memory bandwidth is crucial for performance improvement.
- Memory Access Latency: The latency in accessing data from memory significantly slows down the computational process, leading to inefficient utilization of computational resources and increased training and inference times. For large-scale AI models, both high memory bandwidth and low latency are essential for performance enhancement.
- Multi-modal Data Processing: AI model tasks often require the simultaneous processing of various types of data, including visual, auditory, and textual data. This necessitates that computing chips possess strong parallel computing capabilities and high memory bandwidth to support the concurrent processing of multiple modalities.
- Intermediate Feature Storage and Exchange: AI model tasks involve extensive storage and exchange of intermediate features, placing higher demands on memory access patterns. Traditional memory architectures may not efficiently support the fast read/write operations required to handle large volumes of features, leading to performance bottlenecks.

### 2.2. Computational and Bandwidth Requirements of AI Models on Computing Chips

**Figure 2:**
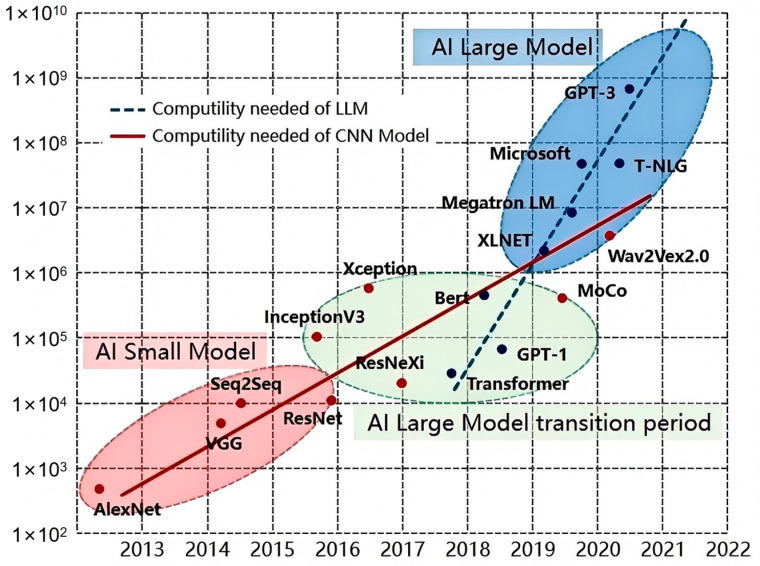
The trend of increasing parameter size in AI models.

With the rapid development of deep learning technologies, AI models are increasingly applied in various fields, including computer vision and natural language processing. The complexity and scale of these models continue to grow, as shown in Figure 2, imposing higher demands on the computility and bandwidth of computing chips. Different AI models used in various applications have distinct requirements for the computility and bandwidth of computing chips.

For example, ResNet (Residual Network) is a classic deep convolutional neural network widely used for image classification tasks. ResNet addresses the vanishing gradient problem in deep networks by introducing residual blocks, allowing for the construction of deeper network structures while maintaining training stability. The ResNet50 model has a parameter size of approximately 25 MB and requires 8.2 GOPs of computility per frame. It involves a large number of convolutional operations and matrix multiplications, which place high demands on computational resources. In initial experiments, the computational-to-bandwidth ratio was normalized for the standard LPDDR4 interface and a specific processor. After normalization, the system configuration had a computility of 12.8 GOPs and a memory bandwidth of 0.03 GB/s in an FPGA environment. Assuming a 70% utilization rate for both computility and bandwidth (an ideal scenario for convolutional neural networks), the theoretical output FPS for the ResNet50 model was 1.09 FPS. However, due to memory bandwidth limitations, the actual output performance (with 70% computility utilization) was only 0.65 FPS. During training and inference, the ResNet model frequently transfers large amounts of data between memory and computing units, including input images, intermediate feature maps, and model parameters. Data transfer accounts for about 8.2% of the total time. Traditional memory systems, such as DRAM, have limited bandwidth, which can become a bottleneck when transferring large amounts of data between memory and computing units, thereby constraining system performance and preventing the processor from achieving its ideal computility. To overcome memory bandwidth limitations, high-bandwidth memory technologies, such as HBM (High-Bandwidth Memory), were employed. After increasing the memory bandwidth (normalized), in an FPGA environment with a computility of 12.8 GOPs and a memory bandwidth of 0.4 GB/s, the performance was no longer limited by memory bandwidth, and the output performance of the ResNet50 model reached 1.05 FPS. On the other hand, the Llama model is a large-scale language model widely used for natural language processing tasks, such as text generation and machine translation. The Llama2-Tiny model has a parameter size of approximately 110 MB and requires 0.22 GOPs of computility per frame. It involves a large number of matrix multiplications and activation function computations. In an FPGA environment with a computility of 12.8 GOPs and a memory bandwidth of 0.03 GB/s, assuming a 70% utilization rate for both computility and bandwidth (an ideal scenario for Transformer network structures), the theoretical output FPS for the Llama2-Tiny model was 4.7 FPS. However, due to memory bandwidth limitations (primarily) and the sequential nature of the Transformer algorithm, the actual output performance (with 30% computility utilization) was only 0.223 FPS. During training and inference, the Llama model frequently transfers large amounts of data between memory and computing units, including input sequences, intermediate features, and model parameters. Data transfer accounts for about 37.44% of the total time, significantly higher than that of image processing applications like the ResNet model. The standard LPDDR4 interface’s bandwidth becomes a performance bottleneck for the entire computing system. By increasing the bandwidth, after normalization, in an FPGA environment with a computility of 12.8 GOPs and a memory bandwidth of 0.4 GB/s, the performance was no longer limited by memory bandwidth, and the output performance reached 4.65 FPS, a 20-fold improvement.

**Figure 3:**
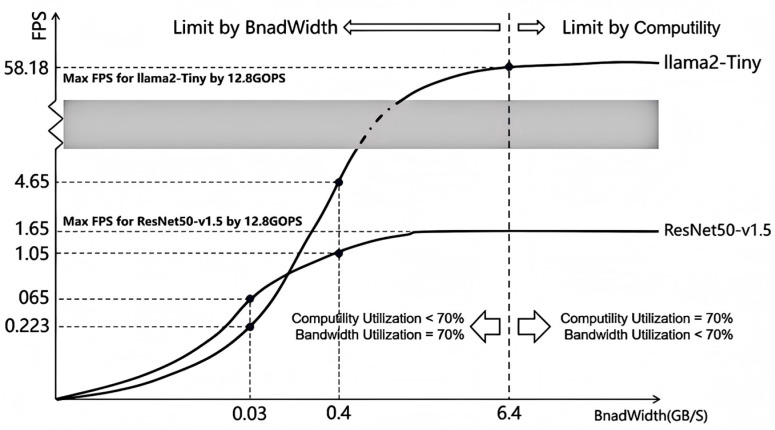
Performance bottlenecks of different AI models in the same hardware.

In conclusion, Figure 3 illustrates that different AI models have varying demands on the computility and bandwidth of computing chips. The ResNet model, primarily involving a large number of convolutional operations and matrix multiplications, places high demands on both computility and bandwidth. The Llama model, involving a large number of matrix multiplications and activation function computations, similarly has high demands on computility and bandwidth. To meet these demands, high-performance computing hardware and high-bandwidth memory technologies are essential to achieve higher processing speeds and system performance.

### 2.3. Data Parallelism and Model Parallelism in AI Models

To handle large-scale AI models, training and inference typically rely on two parallel strategies: data parallelism and model parallelism. Data parallelism involves distributing the data-set across multiple computing nodes, allowing each node to independently process a portion of the data-set. This approach leverages the parallel processing capabilities of multiple nodes, significantly accelerating the training process. By dividing the data-set into multiple subsets and processing these subsets in parallel on different computing nodes, data parallelism effectively utilizes multi-core processors and distributed computing resources, enhancing overall training efficiency. On the other hand, model parallelism refers to partitioning the model itself and distributing the partitions across multiple nodes. This is necessary because large-scale models often exceed the memory and computational capacity of a single node. Model parallelism achieves this by assigning different parts of the model to different computing nodes, enabling the processing of models that surpass the capabilities of a single node. For example, certain layers of a deep neural network can be processed on one node, while other layers are processed on another node, thus achieving load balancing and efficient resource utilization.

Both data parallelism and model parallelism require efficient communication and synchronization mechanisms to ensure effective parallel processing. Data parallelism necessitates frequent communication of gradients and model updates among the nodes to maintain consistency. If communication is untimely or inaccurate, it can lead to non-convergence or performance degradation in model training. Therefore, efficient communication mechanisms are crucial for data parallelism, and common communication mechanisms include All-Reduce and parameter servers (Parameter Server). Model parallelism, meanwhile, requires coordinated data exchanges between nodes to ensure seamless collaboration of different parts of the model. For instance, during forward and backward propagation, intermediate results and gradient information need to be exchanged between different nodes. Efficient synchronization mechanisms can reduce communication latency and improve the performance of model parallelism. Common synchronization mechanisms include synchronous and asynchronous update strategies, as well as various optimization algorithms, such as gradient accumulation and mixed precision training. Hybrid parallel processing combines the advantages of data parallelism and model parallelism to address the challenges of training and inference for large-scale AI models. By leveraging the strengths of each approach, hybrid parallel processing can achieve higher scalability and efficiency. For example, in training large-scale deep neural networks, data parallelism can be used initially to accelerate the training process, and then model parallelism can be introduced when the model size exceeds the capacity of a single node. This hybrid strategy not only maximizes the utilization of computational resources but also effectively addresses limitations in memory and computational capacity, becoming a key strategy for enhancing the performance of AI systems.

## 3. Motivation

### 3.1. Deficiencies of PIM in AI Model Applications with Other Mature Storage Media

Processing in memory (PIM) technology, which integrates computational functions into storage devices, reduces data transfer latency and power consumption, thereby improving processing efficiency. However, there are still some deficiencies or challenges in applying PIM to large-scale AI models using different storage media, and these deficiencies can vary depending on the specific storage medium used.

SRAM, the primary embedded memory in current logic processes, is available in any standard process and logic node. At the circuit level, it offers the fastest read/write and operational speeds among all storage media, making it highly adaptive for applications requiring quick responses, such as autonomous driving and real-time decision systems. However, SRAM, as an on-chip embedded cache, has a very limited storage capacity due to the complexity of its storage unit circuit structure (at least six transistors). For contemporary AI models like the Llama2 model, a single inference requires loading 70 GB (INT8) of parameters. Even the Groq Software-defined Tensor Streaming Multiprocessor [13], which has 230 MB of ultra-large cache, needs to update the on-chip parameter data at least 300 times. While Groq clusters currently outperform NVIDIA’s solutions in low-latency inference scenarios, the memory constraints of the Groq architecture pose significant challenges for large-scale language models. Specifically, each Groq card has 230 MB of SRAM, and a cluster of 576 cards provides a total of 100 GB of SRAM [13]. After reserving memory for model weights, only about 30 GB remains for storing activation values and KV-Cache. For the Llama2 70 B model, which uses the Grouped Query Attention (GQA) algorithm, each layer requires storing eight attention heads, with the KV-Cache size for each token being 8 × 128 (head dimension) × 80 (sequence length) × 2 (K + V) × 2 (bytes) = 320 KB. Given the remaining 30 GB of memory, this can store approximately 90 K tokens. This 90 K token capacity must be shared among all concurrent requests, limiting the maximum context length for a single request to less than 100 K tokens. Long-context scenarios, which are receiving increasing attention in current research and applications, clearly require more capacity. Long-context models need larger context lengths to capture complex dependencies and maintain consistency over long sequences. Therefore, the pure SRAM memory constraint of the Groq cluster poses a significant obstacle to supporting these advanced models in practical applications.

Flash memory demonstrates certain advantages in PIM architectures, such as its non-volatile characteristics and potential for energy savings in certain fields. However, it also faces a series of challenges and limitations. First, Flash memory has relatively slow write and erase operations, particularly the erase process, which can hinder applications requiring frequent data updates. Second, the manufacturing process for Flash memory has reached its limits at 28–40 nm, making it difficult to further reduce device size to improve integration and reduce costs. Additionally, the density of Flash memory cells is only about one-third that of SRAM, limiting its competitiveness in high-density storage solutions. Furthermore, due to its specific market positioning and limited application scenarios, it has not gained widespread recognition in broader markets. Lastly, while the scale of Flash PIM has seen significant growth compared to SRAM, its scalability remains limited, especially in real-time data processing and high-throughput applications. Despite its potential in PIM architectures, the inherent technical limitations and market adaptability issues of Flash memory present numerous challenges to its widespread adoption and application.

Several emerging storage media, such as MRAM, RRAM, and PCRAM, show potential in PIM architectures but come with significant limitations.

- MRAM (Magnetic Random Access Memory): MRAM has high manufacturing costs and low yields, especially at advanced process nodes. It also has slower write speeds, limited storage capacity, and poor compatibility with existing CMOS processes.
- RRAM (Resistive Random Access Memory): RRAM has a complex physical mechanism, leading to performance constraints. It has high manufacturing costs, low yields, and poor durability, with insufficient market validation.
- PCRAM (Phase Change Random Access Memory): PCRAM has a complex physical mechanism and subpar performance, with the worst speed, power consumption, and endurance among emerging storage technologies. Its poor availability and limited research and application efforts further hinder its adoption.

### 3.2. Limitations of the Previous DRAM-Based PIM Research

Recently, Samsung Electronics released the HBM-PIM chip based on HBM2 technology. HBM-PIM can leverage an internal bandwidth of 1 TB/s, which is four times higher than the external bandwidth of 256 GB/s. Additionally, HBM-PIM achieves a 3.5- to 11.2-times performance improvement in neural network applications such as DS2, GNMT, and AlexNet with only a 5.4% increase in power consumption. SK Hynix also introduced the GDDR6-AiM PIM chip based on GDDR6 technology [9,14,15]. Unlike HBM-PIM, GDDR6-AiM extends the functionality from the existing JEDEC standards by adding several new commands, enabling it to accelerate activation functions not supported by Samsung’s HBM-PIM. In addition to all operations paired with memory banks, each processing unit in GDDR6-AiM can exchange data with other units through a global buffer added to the AiM controller on the host chip. However, these data exchanges are coordinated by the host, and each processing unit cannot independently access remote banks. As a result, GDDR6-AiM achieves a throughput of 1 TFLOPS at bfloat16 precision, representing a 16.64-times performance improvement over the Intel Xeon Gold 6230 in GPT-3.

These independent 3D-stacked DRAM studies propose a similar design where each processing unit can independently execute read/write memory accesses to each memory bank without memory command synchronization [16,17,18]. Although asynchronous PIM execution is an effective method for optimizing parallel tensor cores, this approach requires significant changes to the interface between the host and DRAM chips. In the current DRAM interface, the host CPU or GPU sends requests to the memory banks via a memory controller. However, current DRAM-PIM research assumes that the memory controller is integrated independently into each PIM’s logic module. This means that independent PIM designs can only be realized through a complete overhaul of the interface between the host and DRAM. Due to the reluctance of CPU and GPU manufacturers to change memory interfaces and offload their computational capabilities to DRAM, the likelihood of a full redesign of DRAM interfaces for PIM is very low. This is primarily because existing memory interfaces have been optimized to efficiently support current computational demands, and a complete redesign could introduce uncertainties in compatibility and performance. Therefore, while independent PIM designs hold theoretical potential, they face numerous challenges in practical implementation.

## 4. The Architecture of SMPU

### 4.1. Overall Architecture with 3D Computing Cluster–DRAM Face-to-Face Hybrid Bonding

**Figure 4:**
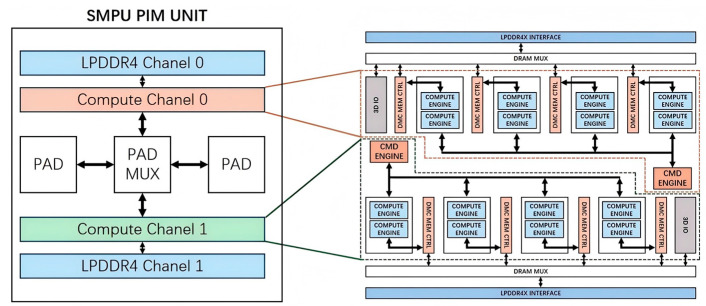
SMPU architecture.

The entire chip is composed of a DRAM chip fabricated using 25 nm technology stacked on top of a computing cluster chip fabricated using 55 nm technology. These two parts are connected via uniformly distributed copper metal pads, ensuring efficient signal transmission and data exchange. The DRAM and the computing cluster logic have the same dimensions, allowing them to align perfectly in the physical layout, maximizing space utilization and performance. As shown in Figure 4, the compute section consists of two channels, each equipped with an instruction processing engine responsible for instruction scheduling and four compute engine blocks designed for large-scale data operations. Each compute engine block contains two sub-compute engines, and each sub-compute engine corresponds to one physical memory block. Each memory block is divided into four banks, with each bank having a capacity of 4 MB. The area of each block is a specific value, and the total capacity of the dual-channel memory is 0.5 GB. This design not only provides ample storage space but also ensures efficient data management and access.

The memory controller for each DRAM block is located in the corresponding position within the computing cluster, naturally dividing the computing cluster into eight blocks, with each compute engine block aligned with one DRAM block. This one-to-one mapping simplifies data access paths, reduces latency, and enhances data transfer efficiency. Each compute engine in the computing cluster can directly access its corresponding DRAM block and can also access all other memory blocks via the on-chip bus. This flexible access mechanism allows compute engines to quickly retrieve and process data as needed, supporting efficient parallel computation. Through block-aware design and placement strategies, the compute engines on the computing cluster chip can span multiple logical blocks and access multiple DRAM blocks, further enhancing the system’s flexibility and scalability. The 0.5 GB DRAM blocks can serve as both memory and cache for the compute cluster engines. In dual-channel mode, each channel has one instruction processing engine and eight compute engines. This design enables each compute engine to efficiently access its local DRAM block and, through the on-chip bus, access the DRAM blocks of other compute engines, facilitating efficient data sharing and parallel processing.

### 4.2. Dual-Channel Memory Address Space and Bus Within the SMPU

**Figure 5:**
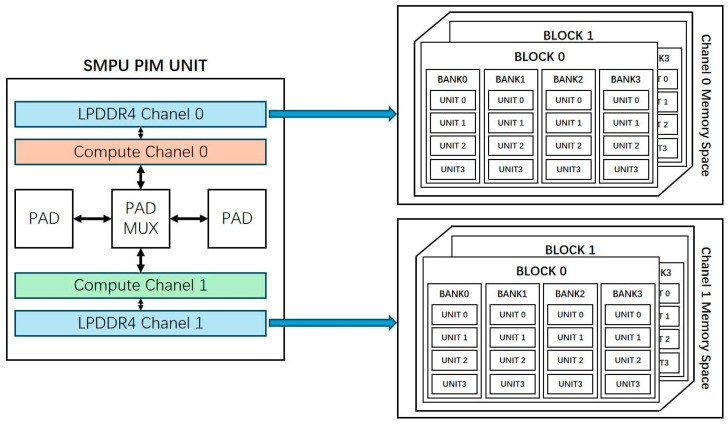
Dual-channel memory address space in SMPU.

In Figure 5, the memory within each SMPU particle is divided into two channels. Each channel’s memory space is evenly divided into eight blocks based on four compute engine blocks, with each compute engine directly corresponding to one DRAM block in the physical layout. Each memory block is further subdivided into four different banks, with each bank granularized into four groups of 4 MB memory address spaces, allowing for more flexible parameter deployment and memory allocation. From memory space allocation to model deployment, the two channels can operate independently or be interrelated. The dual-channel design within each SMPU particle plays a crucial role in data access and model deployment. When the SMPU performs dual-channel data access, the working mode between the two channels is similar to the dual-channel mode on a motherboard. The processors within the SMPU can communicate simultaneously with two independent memory channels, theoretically doubling the total bandwidth within the SMPU and significantly improving data read/write efficiency.

This parallel communication method not only avoids data contention and bottlenecks associated with the single-channel mode but also optimizes system performance through load balancing. This is particularly suitable for high-bandwidth and low-latency applications such as high-performance computing, real-time data analysis, and image processing.

**Figure 6:**
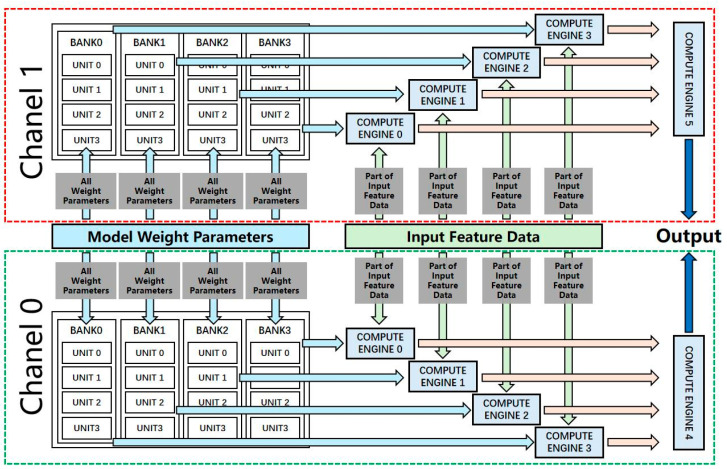
Single-modal model data parallelism.

**Figure 7:**
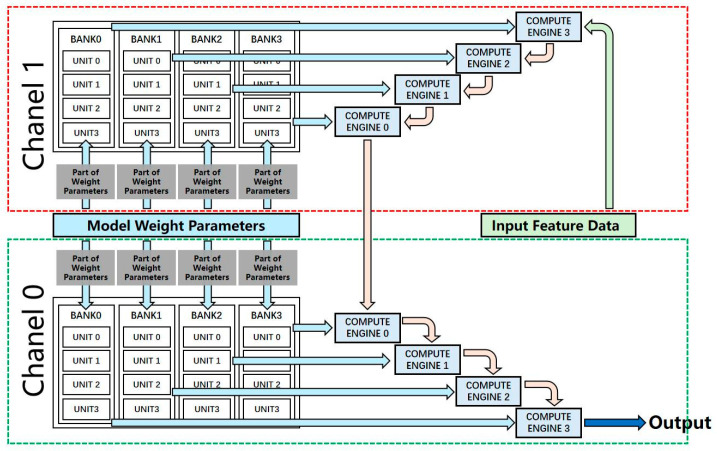
Single-modal model operator parallelism mode.

**Figure 8:**
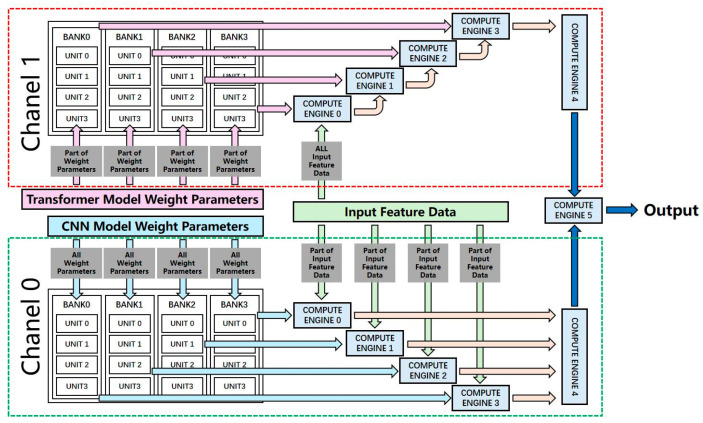
Dual-modal model parallelism.

When the SMPU runs models in dual-channel mode, it supports three modes: single-modal model data parallelism (Figure 6), single-modal model operator parallelism (Figure 7), and dual-modal model parallelism (Figure 8). In single-modal model data parallelism mode, the SMPU divides the input data-set into multiple subsets and distributes these subsets in parallel across all 32 banks. Each bank stores the same weight parameters and processes its own subset of data independently. During training, model parameters are synchronized through gradient aggregation and parameter updates. During inference, the results from each processing unit are combined to produce the final output. In single-modal model operator parallelism mode, the SMPU divides the weight parameter data according to the model layers and sequentially distributes these subsets across all 32 banks in the two channels. The input data-set is processed in a pipeline fashion, with the computation results from each bank being transmitted to the next bank corresponding to the next model layer for further computation. In dual-modal model parallelism mode, the two channels operate independently, with their respective instruction scheduling engines configured and controlling the instruction flow to run different AI models. For the same input data-set, different application scenarios can be processed simultaneously in different channels. For example, for image input, the two channels can perform image recognition and image semantic analysis separately, outputting text descriptions. Additionally, one channel (channel 1) can handle speech-to-text conversion, while the other channel (channel 0) can perform semantic analysis on the resulting text and output inference results.

### 4.3. Dual-Mode Compute Memory Controller

To enable mode switching between host memory and compute memory, we integrated a new memory space allocation and parsing controller, called the Dual-Mode Compute Memory Controller, within each bank of the SMPU memory particles. The Dual-Mode Compute Memory Controller is responsible for data routing and address mapping across three distinct paths: (1) Host Access to DRAM Main Memory Space: This path allows the host to access and write data to the main memory space in DRAM. (2) Host Configuration of PIM Core Parameter Registers: This path enables the host to configure the parameter registers of the PIM core. (3) PIM Core Access to DRAM Compute Memory Space: This path allows the PIM core to access and write data to the compute memory space in DRAM.

**Figure 9:**
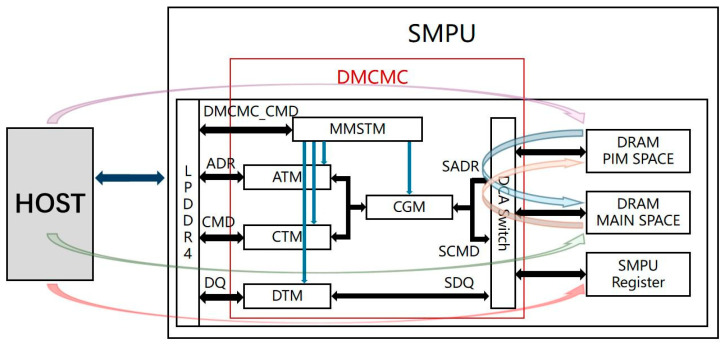
SMPU communication structure by DMCMC.

Within the Dual-Mode Compute Memory Controller, the Address Translation Module (ATM), Command Translation Module (CTM), and Command Generation Module (CGM) handle the address mapping for the three different paths (see Figure 9). The Data Translation Module (DTM) manages direct data transmission between DDR, internal data buses, and DRAM. The Multi-Mode State Machine (MMSTM) switches the state machine according to different operating modes to generate the corresponding control flow. The SMPU operates in two primary modes: Memory Mode and PIM Mode.

*A*. *Memory Mode (Single-Bank Mode)*

In this mode, the SMPU behaves like a conventional DRAM, allowing the host to access the main memory. The host chip accesses individual banks using standard memory commands and addresses, with each memory request targeting a specific bank. This mode is primarily used for traditional memory read and write operations and is suitable for most regular application scenarios. In Single-Bank (SB) mode, the SMPU behaves identically to traditional DRAM, ensuring compatibility with existing systems.

*B*. *PIM Mode*
  (1) *All-Bank (AB) Mode*

This is a special mode that allows the host chip to access all banks in a channel simultaneously through a single memory transaction. In this mode, memory commands, row addresses, and column addresses issued by the host chip are broadcast to all banks, enabling synchronous operations. AB mode is mainly used for configuring and programming PIM cores, allowing the host to write static weight data and program the same PIM core to all processing units in parallel, thereby improving configuration efficiency.

(2) *All-Bank PIM (AB-PIM) Mode*

This is the core mode of the SMPU, used for executing actual PIM (in-memory computing) cores. In AB-PIM mode, each memory transaction not only accesses all banks but also executes the programmed PIM cores in parallel. This means that in a single memory transaction, all processing units in the banks execute the same computational task simultaneously, significantly enhancing computational efficiency and parallelism.

The mode switching process for the SMPU begins with the initial state in Single-Bank (SB) mode, where the SMPU behaves like a regular DRAM and handles memory requests from the host. To enter All-Bank (AB) mode, the host chip sends a series of memory commands through the Dual-Mode Compute Memory Controller, switching the SMPU from SB mode to AB mode, allowing the host to program the PIM cores to all processing units in parallel. After inserting PIM core instructions, the host sends another set of memory commands to switch the SMPU from AB mode to All-Bank PIM (AB-PIM) mode, where each memory transaction will execute the programmed PIM cores in parallel. Once the kernel execution is complete, the host sends a sequence of memory commands to switch the SMPU back from AB-PIM mode to SB mode, restoring normal memory access mode and allowing the system to continue handling regular memory requests.

## 5. Evaluation Methodology and Experimental Setup

To rigorously evaluate the proposed SMPU architecture without relying on high-level software simulators (which often require abstract assumptions regarding compute utilization and memory contention), we employ a cycle-accurate FPGA-based RTL simulation and execution framework. This approach ensures that the control logic, dataflow routing, and pipeline stalls are modeled at the exact hardware clock-cycle level. The absolute performance and power metrics are then derived by mapping the FPGA cycle counts to the target ASIC/3D-stacked technology node.

### 5.1. Baseline and Proposed System Configurations

We evaluate the SMPU by comparing it against a standard baseline computing system to quantify the architectural benefits:

(1) Baseline System: A high-performance host processing platform (XPU, comprising a general-purpose CPU and a dedicated Neural Processing Unit (NPU)) equipped with standard off-chip DDR4 DIMMs. The memory controller handles standard read/write requests, and data must traverse the limited-bandwidth external DDR bus to reach the host’s local caches and the NPU compute units.
(2) Proposed System (SMPU): The identical host XPU integrated with the proposed SMPU. The SMPU replaces the standard memory modules, featuring the Dual-Mode Compute Memory Controller and the 3D-stacked dual-channel computing clusters. Data processing is offloaded to the logic layer, leveraging the high-bandwidth hybrid bonding interconnects, while the host XPU manages control flows and complex post-processing tasks.

### 5.2. Cycle-Accurate RTL Execution and Hardware Modeling

The entire SMPU data-path, including the Dual-Mode Controller, the Network-on-Chip (NoC), and the many-core computing clusters, is implemented in Verilog and executed on a Xilinx UltraScale+ FPGA platform using a cycle-accurate RTL simulation environment.

Memory Timing and Bandwidth Model: Since physical 3D-stacked DRAM is not available, the DRAM behavior is modeled within the RTL testbench using parameterized latency counters. We strictly enforce JEDEC-compatible timing parameters (e.g., tRCD, tCL, tRAS, and refresh intervals) corresponding to DDR4. The internal bandwidth of the SMPU (0.25 TB/s per channel) is modeled in the RTL by configuring a wide internal data-path (1024-bit) and the simulated clock frequency, ensuring that memory contention and bank conflicts naturally stall the pipeline when bandwidth limits are reached.

Computational Utilization: Unlike analytical simulators that assume a fixed MAC (Multiply–Accumulate) utilization rate, our RTL implementation inherently captures the real computational utilization. Pipeline stalls caused by data dependencies, NoC routing delays, and memory access bottlenecks are executed cycle-by-cycle. Therefore, the reported speedups and utilization rates are the direct, emergent results of the hardware execution, reflecting realistic hardware constraints rather than idealized software assumptions.

### 5.3. Technology Scaling to Target ASIC/3D Node

While the FPGA platform provides ground-truth cycle counts and functional validation, FPGA fabric (LUTs/BRAMs) operates at lower frequencies and consumes significantly more power/area than custom silicon. To report realistic metrics for the proposed 3D-stacked system, we translate the FPGA results to a target 28 nm technology node using the following methodology:

Execution Time (Performance Scaling): The total execution time for AI workloads (e.g., ResNet50, Llama) is calculated by dividing the total clock cycles captured from the cycle-accurate FPGA execution by the target ASIC clock frequency. Based on the logic depth of our RTL design, the target frequency for the SMPU logic layer is conservatively set to 500 MHz. Pipeline stalls and memory access latencies are adjusted proportionally during this scaling.

Area and Power Estimation: FPGA power reports heavily overestimate ASIC power due to programmable routing overhead. Therefore, we do not use direct FPGA power measurements. Instead, we employ established analytical modeling tools and literature-based scaling factors. The logic area and dynamic/leakage power of the Dual-Mode Controller and compute clusters are estimated using McPAT and Aladdin frameworks scaled to the 28 nm node. The DRAM array power, 3D-bonding interconnect power, and refresh overhead are calculated using the CACTI-P tool. This methodology ensures accurate, sign-off-equivalent estimations without the overestimation inherent in raw FPGA fabric reports.

## 6. The FPGA Simulation of SMPU Architecture

### 6.1. FPGA System Architecture

**Figure 10:**
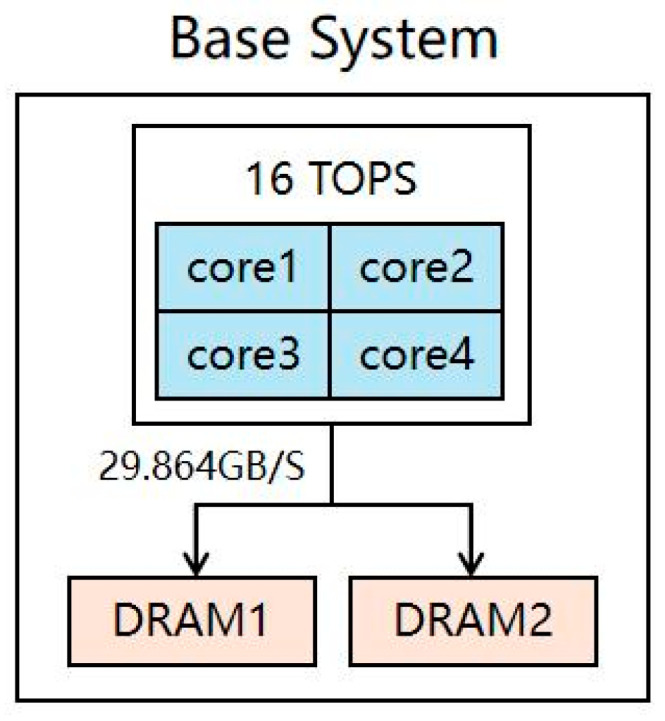
Base system architecture in FPGA.

**Figure 11:**
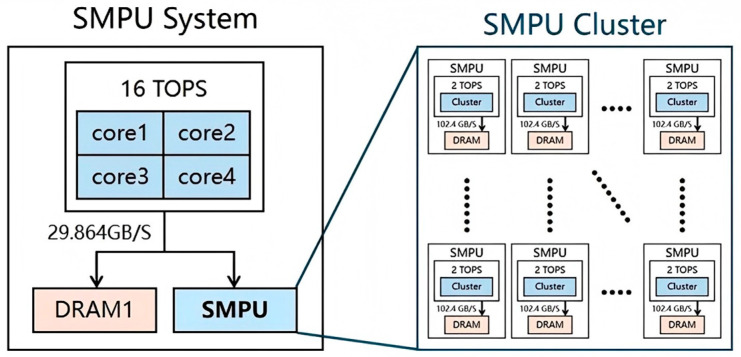
SMPU system architecture in FPGA.

To validate the correctness and advanced nature of the SMPU (Symmetric Multi-Processing Unit) architecture before tape-out, we conducted an FPGA simulation comparison between a baseline system and an SMPU system. We configured two different hardware setups: one simulating the Von Neumann baseline system (Base System: XPU + 2 × DRAM, see Figure 10) and the other simulating the in-memory computing SMPU system (SMPU System: XPU + DRAM + SMPU, see Figure 11). The hardware configuration of the baseline system was set to a computility of 16 TOPS and a memory bandwidth of 29.864 GB/s. For the SMPU system, the hardware configuration (per particle) was set to a computility of 2 TOPS and a memory bandwidth of 102.4 GB/s. We deployed both systems on the FPGA and tested the performance of seven AI models: ResNet50-v1.5, RetinaNet, RNNT, ChatGPT-J 7B, Llama 7B, Llama 8B, and DLRM.

The performance metrics included output performance (FPS), bandwidth utilization, computational utilization, memory access power consumption, and system power consumption. In the FPGA, we set up two comparison systems, as shown in the figure, to evaluate the performance and power consumption improvements of the system equipped with SMPU compute memory. The baseline system includes one compute core and two memory modules. By adjusting the number of MAC units and the system frequency, the baseline system was configured with a core computility of 16 TOPS and a memory bandwidth of 29.864 GB/s (LPDDR4). The SMPU system replaces one of the memory modules in the baseline system with SMPU compute memory. The SMPU compute memory is configured with a single particle computility of 2 TOPS, a single particle capacity of 0.5 GB, and the ability to deploy 1 to 16 SMPU compute memory particles based on the parameter size requirements of the models.

### 6.2. Primary Benchmark with Dual-Channel Hybrid Parallel Deployment

*A*. *Vision Model: ResNet50*

**Figure 12:**
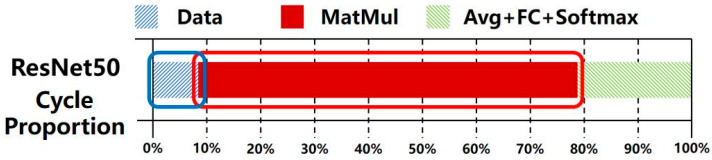
The proportion of time consumed by each part of ResNet50.

ResNet50 is a deep convolutional neural network that addresses the vanishing gradient problem in deep neural networks through the introduction of residual blocks (Residual Blocks). This innovation allows the network to be deeper while maintaining high training efficiency and model performance. During each frame output process in ResNet50, approximately 8% of the time is spent on data transfer between the computing engine and DRAM, while nearly 70% of the time is devoted to linear matrix computations within the network as revealed by Figure 12.

When performing convolutional calculations on a feature input matrix in each layer of ResNet50, the same set of convolutional kernels is used on the same feature input matrix. The convolutional kernels slide over the feature matrix in a windowed manner to produce the convolutional output for that layer. The computational results from each window-sliding operation within the same layer are not temporally correlated. Therefore, for the convolutional computations in the same layer, the identical weight data (i.e., convolutional kernels) can be simultaneously deployed in each bank of two channels. The feature input matrix can be partitioned into corresponding sub-matrices and fed into different computing engines for large-scale parallel convolutional computations, thereby achieving data parallel deployment in ResNet50.

*B*. *Language Model: Llama2*

**Figure 13:**
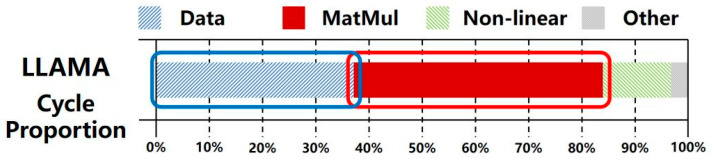
The proportion of time consumed by each part of Llama.

Llama is a large language model (LLM) designed for text processing. It addresses the challenges of deep neural networks by leveraging advanced architectures and training techniques, enabling it to perform a wide range of natural language processing (NLP) tasks with high accuracy and efficiency. During each forward pass of the Llama model, approximately 38% of the time is spent on data transfer between the computing engine and DRAM, while nearly 45% of the time is devoted to linear matrix computations within the network as revealed by Figure 13.

When processing a single input token, the Llama model uses a series of transformer layers, each of which performs self-attention and feed-forward operations. Each transformer layer processes an input feature matrix using the same set of attention heads and feed-forward weights. The attention mechanism involves sliding over the input sequence to generate attention scores, which are then used to compute weighted sums of the input features. The feed-forward operations include applying linear transformations followed by non-linear activation. The computational results from each attention head and feed-forward operation across different layers of the network have strong temporal correlations. Therefore, for the computations in the Llama network, different sets of weight data (i.e., attention heads and feed-forward weights) for each layer can be sequentially deployed in each bank of two channels. The input feature matrix and intermediate computation results from each layer flow through intra-channel and inter-channel data buses and are sequentially fed into adjacent computing engines for linear matrix computations and non-linear calculations in the attention heads, thereby achieving operator parallel deployment in the Llama model.

### 6.3. Performance of FPGA Simulation

*A*. *Performance of FPS*

**Figure 14:**
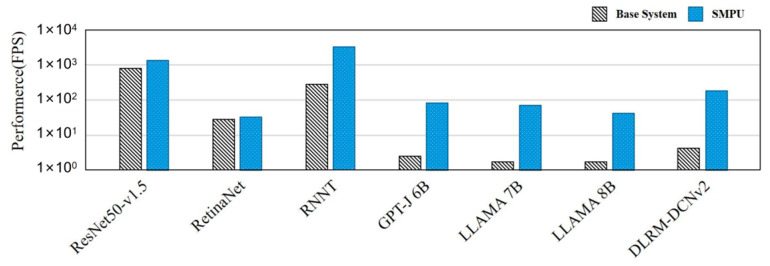
Performance of SMPU and base systems on benchmark applications.

**Figure 15:**
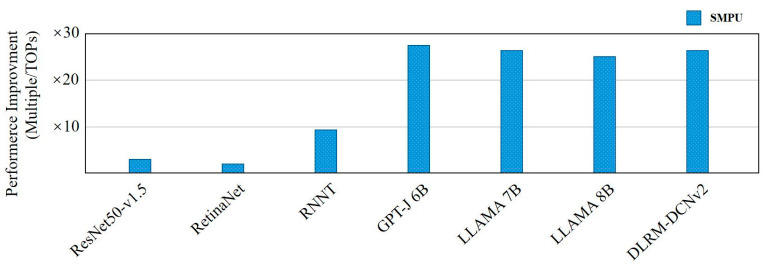
Improvement of SMPU system on benchmark applications.

As shown in Figure 14 and Figure 15, the results of the FPGA simulation experiments demonstrate significant performance improvements of the SMPU (Symmetric Multi-Processing Unit) system across various AI models. Under the same computility configuration, the SMPU system showed substantial performance gains. Specifically, for the ResNet50-v1.5 model, the SMPU system’s performance increased from 943.01 FPS in the baseline system to 1468 FPS, achieving a 1.5× performance improvement. Similarly, the performance of the GPT-J 6B, Llama 7B, Llama 8B, and DLRM-DCNv2 models on the SMPU system improved from 2.61 to 3.48 FPS in the baseline system to approximately 71.68 to 89.57 FPS, resulting in performance improvements ranging from 25.73× to 27.46×.

These results indicate that the SMPU system has a significant advantage in handling large-scale deep learning models. However, for the RetinaNet model, the performance on the SMPU system did not show significant changes compared to the base system, remaining around 48.151 FPS and 47.18 FPS, respectively. This suggests that the SMPU may have reached a performance bottleneck for the specific model.

*B*. *Performance of Power*

**Figure 16:**
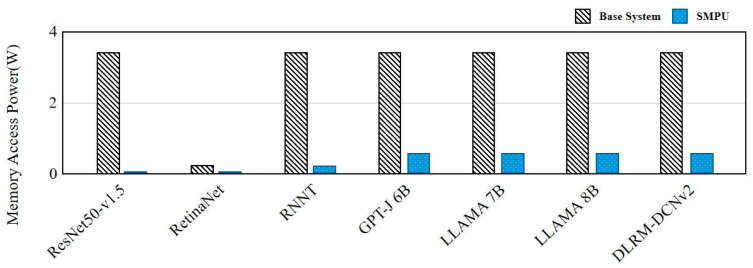
Memory access power of systems on benchmark applications.

**Figure 17:**
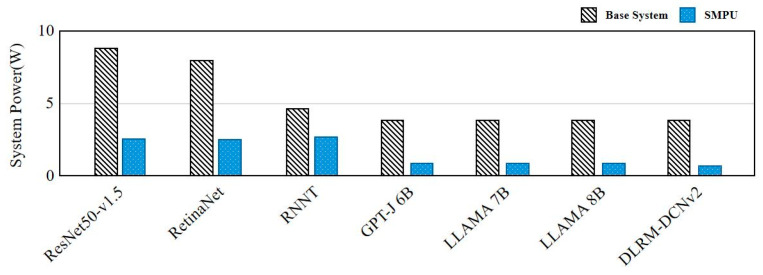
System power of systems on benchmark applications.

As shown in Figure 16 and Figure 17, the power consumption results from the FPGA simulation experiments indicate that the SMPU system significantly reduces memory access power consumption across all tested AI models. The memory access power consumption of the SMPU system remains within the range of 0.05 to 0.2 W for all models. This is because the SMPU system completely eliminates frequent data exchanges between the processor and DRAM, ensuring that data movement and computation occur solely within the memory. Furthermore, the SMPU system employs a weight refinement strategy, which minimizes data overwrites within the SMPU, such that only feature input data and intermediate computation results are written and erased. Consequently, the SMPU system achieves a total power consumption reduction of nearly 80%. In contrast, the baseline system’s memory access power consumption, constrained by frequent data interactions between the host and DRAM, remains consistently at approximately 3.5 W, corresponding to the power consumption at peak bandwidth (70% bandwidth utilization).

*C*. *Performance of Bandwidth and Computational Utilization*

**Figure 18:**
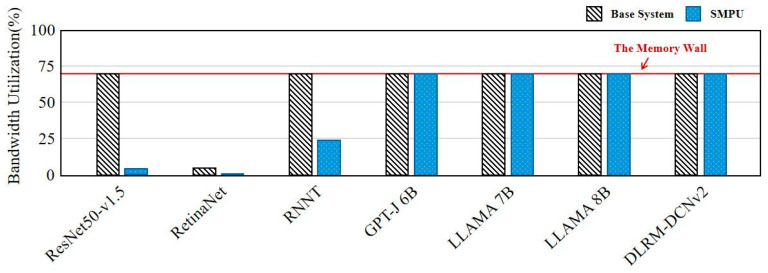
Bandwidth utilization of systems on benchmark applications.

**Figure 19:**
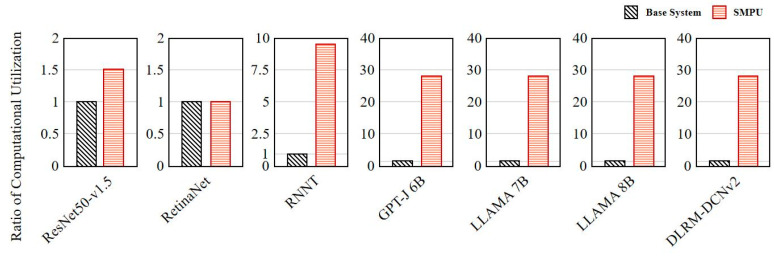
Computility utilization of systems on benchmark applications.

As shown in Figure 18 and Figure 19, under the same computility configuration, the bandwidth allocated to the baseline system is 29.864 GB/s. The results of the FPGA simulation experiments show that, when running most AI models, the system bandwidth remains at full load (70% bandwidth utilization, approximately 21.5 GB/s). This indicates that the system is experiencing a memory wall bottleneck, which limits the system’s output performance.

In the SMPU system, when running models such as ResNet50-v1.5, RetinaNet, and RNNT, the system bandwidth is under light load. This is primarily because these models are compute-sensitive, requiring a higher ratio of computility to bandwidth. Their demand for system bandwidth is much lower than their demand for computility. However, when running large language models and recommendation models, the SMPU system’s bandwidth utilization also reaches 70%, corresponding to the peak bandwidth under memory wall constraints (approximately 73.72 GB/s). This is because these large language models and recommendation models are bandwidth-sensitive, requiring much less computility but significantly more system bandwidth. Therefore, even with the increased system bandwidth in the SMPU system, a memory wall bottleneck still occurs when running these models. From the results, it is evident that even for the ResNet50 model, which is less sensitive to bandwidth, the performance in the baseline system is still constrained by the memory wall.

**Figure 20:**
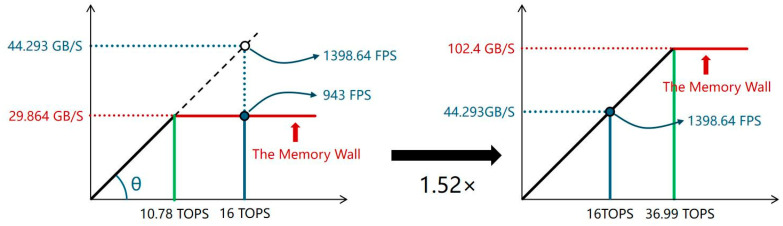
Performance improvement analysis of ResNet50 in two systems.

**Figure 21:**
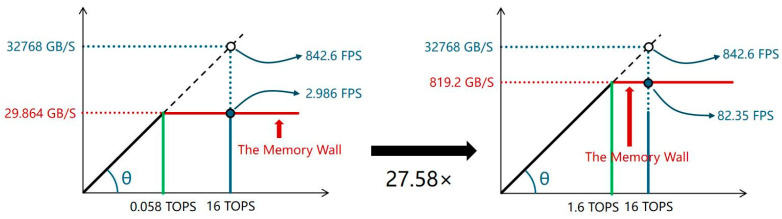
Performance improvement analysis of Llama in two systems.

On the other hand, the increase in system bandwidth in the SMPU system significantly enhances the computility utilization. For compute-sensitive models, the increase in internal memory bandwidth through the SMPU leads to a 1.526 to 9.246× improvement in computility utilization (Figure 20). A detailed analysis is provided in Section 6. For bandwidth-sensitive large language models and recommendation models, the computility utilization is improved by 26.88× (Figure 21).

*D*. *Analysis of Normalized Metrics and Practical Advantages*

As illustrated in Table 1, the proposed SMPU demonstrates highly competitive normalized metrics compared to existing solutions.

First, in terms of Bandwidth Density and Energy Efficiency (TOPS/W), SMPU achieves comparable or superior projected performance relative to commercial HBM-PIM and GDDR6-AiM. This is primarily attributed to the 3D hybrid bonding technology, which provides massive internal bandwidth (2 TB/s) with significantly lower I/O power consumption compared to traditional off-chip high-speed interfaces.

Second, regarding Supported Workloads, unlike early academic PIM designs (e.g., FIMDRAM) that are often restricted to simple vector operations or specific GEMV kernels, the dual-channel many-core computing clusters in SMPU provide ample and flexible compute capacity, enabling the efficient execution of complex, multi-modal AI models like ResNet50 and Llama2.

Most importantly, the unique practical advantage of SMPU lies in its Host Interface Compatibility. While HBM-PIM and GDDR6-AiM deliver high bandwidth, they are inherently tied to specialized, proprietary, and costly HBM/GDDR6 physical interfaces, restricting their deployment to high-end, specialized accelerator markets. In contrast, SMPU achieves high internal bandwidth via 3D stacking while exposing a standard DDR-compatible interface to the host XPU. This allows SMPU to be seamlessly integrated into mainstream, capacity-oriented server environments as standard memory modules, drastically lowering the deployment barrier and system integration costs for practical AI computing.

## 7. Discussion

*A*. *Improvement of the SMPU with computility-sensitive Models*

ResNet50 is an image processing model that belongs to the Residual Network (ResNet) series. This series of models addresses the vanishing gradient problem in deep neural networks by introducing residual blocks (Residual Blocks), enabling the network to be deeper while maintaining high training efficiency and model performance. The parameter size and computational requirements for ResNet50 to process one frame are 22.7 MB and 8.2 GOPS, respectively. Its compute-to-bandwidth ratio (defined as the required computational amount divided by the parameter size) is 369.9, classifying it as a compute-sensitive model.

In the baseline system, the memory interface provides a bandwidth of 29.864 GB/s. Given the compute-to-bandwidth ratio requirement of 369.9, the peak computational capacity achievable at 29.864 GB/s bandwidth can be calculated as 10.78 TOPS. The constrained by the memory interface bandwidth, resulting in only 45.5% hardware computational efficiency.

In the SMPU system, the memory bandwidth is increased to 102.4 GB/s. Under this condition, the theoretical peak computational capacity of the hardware is 36.99 TOPS. Consequently, the SMPU system is not limited by the memory wall and fully utilizes the actual computility provided by the hardware, achieving a performance improvement of 1.52×.

*B*. *Improvement of the SMPU with bandwidth-sensitive Models*

Llama is a text processing model that belongs to the large language model (LLM) series. Trained on extensive text data, the Llama model is capable of performing various natural language processing (NLP) tasks, such as text generation, translation, summarization, and question answering. The parameter size and computational requirements for Llama to process one frame are 7168 MB and 14 GOPS, respectively. Its compute-to-bandwidth ratio (defined as the required computational amount divided by the parameter size) is 2, classifying it as a bandwidth-sensitive model.

In the baseline system, the memory interface provides a bandwidth of 29.864 GB/s. Given the compute-to-bandwidth ratio requirement of 2, the peak computational capacity achievable at 29.864 GB/s bandwidth can be calculated as 0.058 TOPS. The peak computational capacity of the baseline system is severely constrained by the memory interface bandwidth, resulting in only 0.3% hardware computational efficiency. In the SMPU system, eight SMPUs are deployed in parallel to store the model parameters, increasing the memory bandwidth to 2 TB/s. Under this condition, the theoretical peak computational capacity of the hardware is 1.6 TOPS. Although the system is still limited by the memory wall, the increase in memory bandwidth significantly improves the performance of the SMPU system on the Llama model, achieving a 27.58× performance enhancement.

It is worth noting that while the memory bandwidth increases by roughly 13×, the model throughput (FPS) rises by 27.58×. For strictly bandwidth-bound workloads, performance improvement is theoretically expected to scale linearly with (or slightly less than) bandwidth growth. This observed “super-linear” speedup is attributed to the non-linear improvement in effective bandwidth utilization and compute-memory overlapping.

In the baseline low-bandwidth scenario, the compute clusters suffer from severe data starvation. The memory subsystem fails to feed weights and activations at the required rate, causing frequent pipeline stalls and bubbles. As a result, the actual effective bandwidth consumed by the compute units is significantly lower than the peak physical bandwidth of the interface. Conversely, when the SMPU provides a ~13× higher internal bandwidth, the data supply bottleneck is fundamentally resolved. The compute pipelines become fully saturated, maximizing the MAC unit utilization. Furthermore, the abundant bandwidth allows for aggressive prefetching and seamless overlapping of memory accesses with computations (effectively hiding memory latency). Consequently, the effective data throughput experienced by the AI model scales super-linearly relative to the raw physical bandwidth expansion, justifying the 27.58× surge in FPS.

*C*. *Theoretical Decomposition of Performance Contributions*

While the overall evaluation demonstrates significant speedups of the proposed SMPU over the baseline (XPU + standard DRAM), it is essential to theoretically isolate the performance gains attributable specifically to the PIM architectural paradigm, as opposed to the mere expansion of raw hardware resources (bandwidth and compute). Since constructing a traditional non-PIM baseline with an identical 2 TB/s off-chip bandwidth and 16 TOPs compute power within the same power and physical envelope is practically infeasible (as it would require massive, power-prohibitive HBM interfaces), we decompose the contributions theoretically:

1. Contribution of Bandwidth Expansion: A significant portion of the speedup, particularly for memory-bound workloads like Llama2-Tiny, stems from alleviating the “Memory Wall.” The transition from standard DDR bandwidth to the 0.25 TB/s internal bandwidth of SMPU drastically reduces data fetch latency. However, bandwidth expansion alone cannot explain the entirety of the performance and energy-efficiency gains.
2. Diminishing Returns of Added Compute Resources: If the 16 TOPs of compute power were added to the baseline system as a discrete accelerator (without increasing the off-chip memory bandwidth), the performance gain would be marginal. Due to the severe bandwidth limitation of standard DDR, the additional MAC units would suffer from extreme data starvation, resulting in very low computational utilization. This proves that merely scaling up compute resources in a traditional Von Neumann architecture is ineffective for AI workloads.
3. The Core Gain of the PIM Architectural Paradigm: The most distinctive contribution of the SMPU is the elimination of redundant data movement. In the baseline system, massive amounts of weights and activation must be continuously shuttled across the off-chip DDR bus, consuming dominant latency and energy. The SMPU architecture fundamentally shifts this paradigm by executing computations in situ (within or directly adjacent to the memory arrays). This near-data processing not only maximizes the utilization of the expanded internal bandwidth but also drastically reduces the data movement overhead. Therefore, the superior performance and energy efficiency of SMPU are intrinsically driven by the PIM data-flow organization, which unlocks the true potential of the underlying hardware resources.

## 8. Conclusions

We propose a new Super Memory Processing Unit (SMPU) to support more efficient and general in-memory computing. Existing DRAM-PIM implementations suffer from limited computational capabilities due to the shared packaging area between memory cells and computational circuits or because the operator circuits are overly customized. To address this issue, the SMPU system employs Hybrid Bonding technology to 3D-stack DRAM and multi-core computational clusters, achieving large-bandwidth (0.25 TB/s per-bank, 2 TB/s for 8-bank system bandwidth) on-chip data transmission via copper interconnects, effectively breaking the memory wall bottleneck.

In practical systems using FPGA, the SMPU has demonstrated significant acceleration across multiple models. FPGA simulation results show that the system equipped with SMPU improves the performance of ResNet50-v1.5 by 5.1 times and Llama2 by 27.43 times, while reducing system power consumption by 71.6% for ResNet50-v1.5 and 77.8% for Llama2. These results highlight the significant advantages of the SMPU in enhancing the performance and efficiency of computing systems.

## Figures and Tables

**Table 1:** Comparison of SMPU with State-of-the-Art PIM Architectures and AI Accelerators.

Architecture	SMPU (Ours)	HBM-PIM (Samsung)	GDDR6-AiM (SK Hynix)	FIMDRAM (ISCA’21)
**Type**	3D-PIM	In-DRAM PIM	Near-Memory	In-DRAM PIM
**Process Node**	28 nm (Logic)/ 20 nm (DRAM)	10 nm (Logic)/1y-nm (DRAM)	1a-nm (DRAM)	20 nm (DRAM)
**Memory Capacity**	8 GB	16 GB	2 GB	8 GB
**Peak Bandwidth**	2048 GB/s	~307 GB/s	~72 GB/s	~25.6 GB/s
**Energy Efficiency (TOPS/W)**	~12.0 *	~12.0	~11.5	~6.5
**Host Interface**	Standard DDR4	HBM2 (Proprietary)	GDDR6 (Proprietary)	DDR4
**Supported Workloads**	ResNet, Llama, Multi-modal	NLP, Translation	LLM, Recommendation	Vector, GEMV

* Note: Values for SMPU are pre-silicon architectural estimations scaled to the 28 nm node using McPAT and CACTI-P. Energy efficiency for commercial chips is derived from published datasheets and ISSCC/JSSC papers, evaluating INT8 dense operations where applicable.

## Data Availability

Data are contained within this article.
